# An exploratory evaluation of the interaction risk between herbal products and pharmaceutical medicines used concurrently for disease management in Blantyre, Malawi

**DOI:** 10.1080/13880209.2025.2586351

**Published:** 2025-11-19

**Authors:** Kumbukani K. Nyirenda, John Mponda, Ibrahim Chikowe, Esther Kawonga, Nellie Twatasha Gomani Phiri, Mervis Msukwa, Chimota Phiri, Amy L. Roe, Mary F. Paine, Bill Gurley, Hellen Oketch-Rabah, Stefan Gafner, Julie Krzykwa, Constance A. Mitchell, Michelle R. Embry, Syril Pettit, Dallas J. Smith

**Affiliations:** a Department of Pharmacy, Kamuzu University of Health Sciences, Blantyre, Malawi; b AO Alliance, Lilongwe, Malawi; c Partners in Health Malawi, Lilongwe, Malawi; d Equip Group, Lilongwe, Malawi; e Department of Medicine, Queen Elizabeth Central Hospital, Blantyre, Malawi; f Procter & Gamble Healthcare, Cincinnati, OH, USA; g Department of Pharmaceutical Sciences, College of Pharmacy and Pharmaceutical Sciences, Washington State University, Spokane, WA, USA; h National Center for Natural Products Research, School of Pharmacy, University of Mississippi, University, MS, USA; i United States Pharmacopeia, Rockville, MD, USA; j American Botanical Council, Austin, TX, USA; k Health and Environmental Sciences Institute, Washington, DC, USA

**Keywords:** Herbal-drug interactions, botanicals, Malawi, ethnobotanical, diabetes, hypertension, safety, HDI, CYPs

## Abstract

**Context:**

The use of herbal products in Malawi remains widespread and culturally significant, often occurring alongside pharmaceutical medicine treatments. As the burden of non-communicable diseases such as diabetes and hypertension continues to rise, the potential for herbal-drug interactions (HDIs) represents an underexamined public health concern.

**Objective:**

Evaluate the concurrent use of herbal products and pharmaceutical medicines among patients with diabetes or hypertension in a large health care facility in Malawi and identify potential adverse HDIs.

**Materials & Methods:**

An exploratory mixed-methods, cross-sectional study was conducted with 301 patients attending a diabetes or hypertension clinic at Queen Elizabeth Central Hospital in Blantyre, Malawi. Participants self-reported herbal and pharmaceutical use, and a targeted literature review was undertaken to assess potential pharmacokinetic and pharmacodynamic interactions between commonly reported herbal products and prescribed medications.

**Results:**

Participants reported concurrent use of a wide variety of herbal products (e.g., garlic, ginger, okra, lemon, mango, moringa) with prescription medicines (e.g., metformin, insulin, glibenclamide, hydrochlorothiazide, enalapril, amlodipine). While clinical outcomes were not independently verified, literature review findings, in some cases, indicated meaningful potential for HDIs.

**Discussion and Conclusion:**

This study provides foundational data on herbal-pharmaceutical co-use in Malawi and highlights the need for expanded research, improved documentation of herbal use in healthcare settings, and improved education for patients and providers. Integrating awareness of herbal product use into clinical care is essential, and the methodology and findings may inform future hypothesis-driven studies across Africa and other regions where traditional and modern medicine use overlaps.

## 1. Introduction

Non-communicable diseases (NCDs), such as diabetes and hypertension, are the leading causes of global morbidity and mortality, accounting for more than half of the world’s disease burden (WHO 2018). In sub-Saharan Africa, NCDs are rapidly emerging as a public health problem, disproportionately affecting vulnerable populations. The WHO estimates that 77% of NCD-related deaths occur in low- and middle-income countries, and by 2030, deaths from NCDs in Africa are expected to surpass those from communicable diseases and perinatal deaths combined (Li et al. 2022). These impacts harm both patient well-being and the region’s economic development (Li et al. 2022).

As NCDs increase, many patients in low- and middle-income countries rely on herbal products for disease management or to support overall health. Known by many names, such as traditional or botanical medicines, herbal products are deeply rooted in culture, accessible, affordable, and serve as healthcare for more than 80% of the African population (WHO 2019). While herbal product use has long been widespread for a variety of health conditions, what is changing is the context: more patients are now relying on them specifically to address chronic NCDs (Thikekar et al. 2021). For instance, a survey in Nigeria reported that up to 46% of patients with diabetes, 39% of patients with hypertension, and 65% of patients with cancer used herbal products alongside pharmaceutical drugs (Ondieki et al. 2017). Additionally, specific to Malawi there are many reports on the use of herbal products in hospital patients, with rates varying from 17.5%(Mbali et al. 2021) to 80.6% (Hill et al. 2022; Mponda et al. 2024) of patients.

The risk of herbal-drug interactions (HDIs) increases with polypharmacy, posing a public health concern due to potential adverse reactions or reduced drug efficacy (Awortwe et al. 2018; Gurley 2025). Like drug-drug interactions, HDIs can be pharmacokinetic (PK), pharmacodynamic (PD), or a combination. Common PK mechanisms include induction or inhibition of drug metabolizing enzymes, such as the cytochrome P450s (CYPs), or transporters such as P-glycoprotein (P-gp, an ATP-binding cassette efflux transporter), leading to altered drug exposure and effects (Fasinu et al. 2012). Because many herbal products are known to affect CYP or P-gp function, co-consumption with pharmaceutical drugs can result in harmful interactions (Sharma et al. 2022; Bukowska et al. 2025).

Despite the rising NCD rates and herbal products use in low- and middle-income countries, data on HDIs remain limited (Shu and Jin 2023). Malawi has experienced a significant increase in the prevalence of hypertension and diabetes over the past decade. In adults, hypertension and diabetes affect approximately 15–29% and 3% of the population, respectively (Price et al., 2018). Limited availability of prescription medications, equipment, and trained providers hampers treatment of these diseases in the country’s healthcare system (Khuluza and Haefele-Abah 2019). Meanwhile, herbal products remain widely used in Malawi (Mbali et al. 2021; Meke et al. 2017), yet data are lacking about their concurrent use with pharmaceutical drugs.

Based on these knowledge gaps, the purpose of this exploratory study was to unravel the practice of simultaneous use of herbal products and pharmaceutical medicines among patents attending diabetes and hypertension clinics at Queen Elizabeth Central Hospital (QECH) in Blantyre, the largest referral hospital in Malawi. QECH cares for patients from both urban and peri-urban settings and manages a high burden of patients with diabetes and hypertension, rendering this site highly appropriate and relevant to investigate concurrent use of herbal products and pharmaceutical drugs. Understanding the identities of herbs and combination patterns is critical to inform potential HDIs and provide improved patient guidance

## 2. Methodology

### 2.1. Study design

A descriptive cross-sectional exploratory study was conducted that involved collecting qualitative and quantitative data. The study population was restricted to patients who attended a diabetes and hypertension clinic at QECH, were 18 years of age or older, were consuming at least one herbal product, and consented to participate in the study. A convenience sampling method was used to recruit participants in the survey between June 2021 and December 2021. Patients enrolled in the study were voluntarily visiting the clinic for consultation or to collect medications. A total of 301 patients participated in the study.

### 2.2. Ethical considerations

The study protocol and questionnaire were approved by the Institutional Review Board, College of Medicine Research and Ethics Committee (Approval number P.04/21/3305). Interested patients provided their informed consent in writing or verbally if they had low literacy. They were informed that no identifiable information would be collected or shared, and a verbal description of the study aims was provided. All participation was voluntary; no compensation was provided.

### 2.3. Data collection

After obtaining written or verbally informed consent from participants, interviews were conducted in the local language, Chichewa, using a semi-structured questionnaire led by a trained data collector. Responses were translated into English by the data collector for documentation purposes (Supplemental File 1). The solicited information included demographics, such as age and sex, self-reported identity of the herbal product and prescription antihypertensive and/or anti-hyperglycemic pharmaceutical drugs used, self-reported diabetes and blood pressure status, and self-reported assessments of perceived positive or negative health effects following consumption of anti-hyperglycemic or anti-hypertensive pharmaceutical drugs and/or herbal products. All participant datasets were assigned a numerical identifier to record their data electronically, and no personal identifiable information was collected. None of the information provided by the participants was cross-referenced against their medical records or otherwise independently verified. Some participants did not respond to specific questions.

### 2.4. Potential herbal-drug interaction (HDI) assessment

The survey data were used to identify the pharmaceuticals most commonly reported to be taken for diabetes and hypertension, as well as the herbal products most often taken concurrently with these pharmaceutical drugs. Using available published literature and expertise from the author team about the PK and PD of the pharmaceutical drugs or herbal product constituents, these combinations were evaluated for potential HDIs. Literature evaluation was conducted *via* a nonsystematic and non-comprehensive review with the goal of identifying reported HDIs, mechanistic mode of action studies on the herbal products reported in the survey, and any adverse event reports linked to the plants identified. Literature evaluation was conducted *via* PubMed and Google Scholar searches. Search terms typically combined the botanical scientific and common names with keywords such as *herbal–drug interaction*, *cytochrome P450 (CYP)*, *P-glycoprotein (P-gp)*, *diabetes*, *hypertension*, *ADME*, *PK*, and *PD*. Additional related terms were used where relevant. This approach was nonsystematic and non-comprehensive, intended to identify reported HDIs, mechanistic studies, and adverse event reports relevant to the herbal products reported in the survey.

### 2.5. Data management and statistical analysis

Quantitative data were analyzed primarily using descriptive statistics. The names of the herbal products reported by the participants were standardized to facilitate analysis (for example, ‘ginger’ versus ‘ginger root’). Because fewer than 10% of the participants provided responses to the open-ended questions about positive/negative health effects following the pharmaceutical or herbal product therapies reported in their interview, the data were considered insufficient to conduct a comprehensive qualitative analysis. This exploratory study intended to generate descriptive data about the concurrent use of herbal products and pharmaceutical medicines. A formal power calculation was not performed, as the study was not designed to test specific hypotheses. Instead, our goal was to identify both patterns of use and potential HDIs of relevance for future hypothesis-driven studies. The dataset has been made publicly available to enable additional analyses.

## 3. Results and discussion

### 3.1. Survey results

Of the 301 participants, the majority (211) were female (Table 1). Most were aged from 36 to 65 years. One person’s age was unknown but was more than 18 years old. The majority resided in peri-urban locations. All participants had a self-reported diagnosis of diabetes or hypertension, and about one-third of them had both.

**Table 1.** Overview of survey participant demographics.

Survey Participants		*Total*		*Female*		*Male*	
Sex Distribution		301		211		90	

Age Distribution	*Unknown*	*18–25 years*	*26–35 years*	*36–45 years*	*46–55 years*	*56–65 years*	*>66 years*
	1	18	24	63	80	75	40

Self-Reported Location of Residence		*Rural/Village*		*Peri-Urban*		*Urban*	
		39		240		22	

Self-Reported Health Diagnosis		Diabetes		Hypertension		Diabetes and Hypertension	
		222		176		99	

Although this study was conducted at a diabetes and hypertension clinic, participants were asked to report on *all* pharmaceutical drugs that they were currently taking. A total of 44 different pharmaceutical drugs were reported and were classified according to therapeutic class (Table 2).

**Table 2.** Frequency of reported use of pharmaceutical drugs by study participants categorized by therapeutic class.

Therapeutic Class	Number of Participants Reporting Current Use
Anti-hypertensives	160
Diabetes management	220
Anti-retroviral	31
Anti-depressants	24
Analgesics	11
Antibiotics	3
Vitamins	2
Other (indications with fewer than 1% reporting use): asthma, clotting disorder, cancer, hyperlipidemia, epilepsy	1

**Figure 1.**
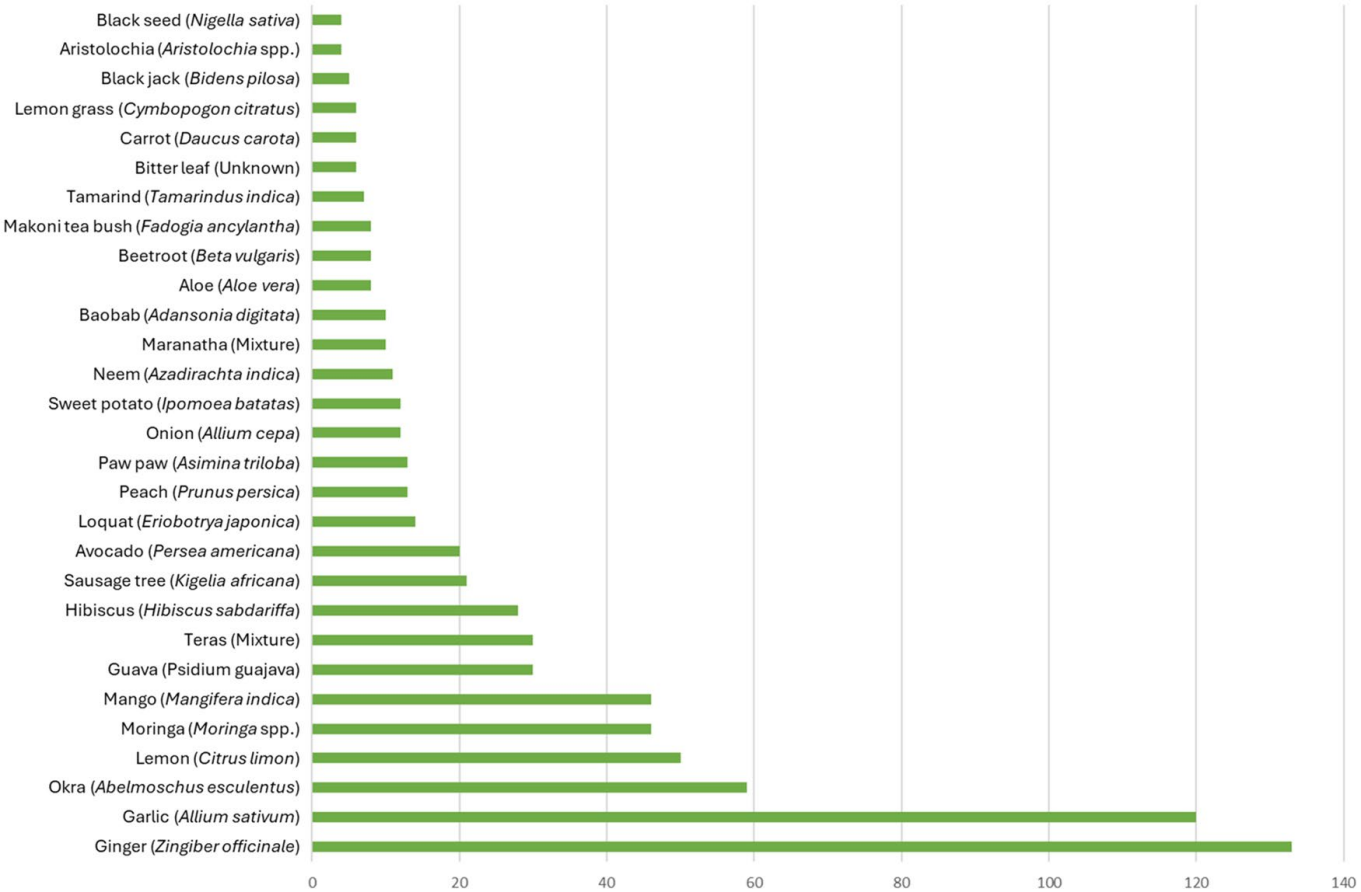
Most commonly reported herbal products by study participants.

**Figure 2.**
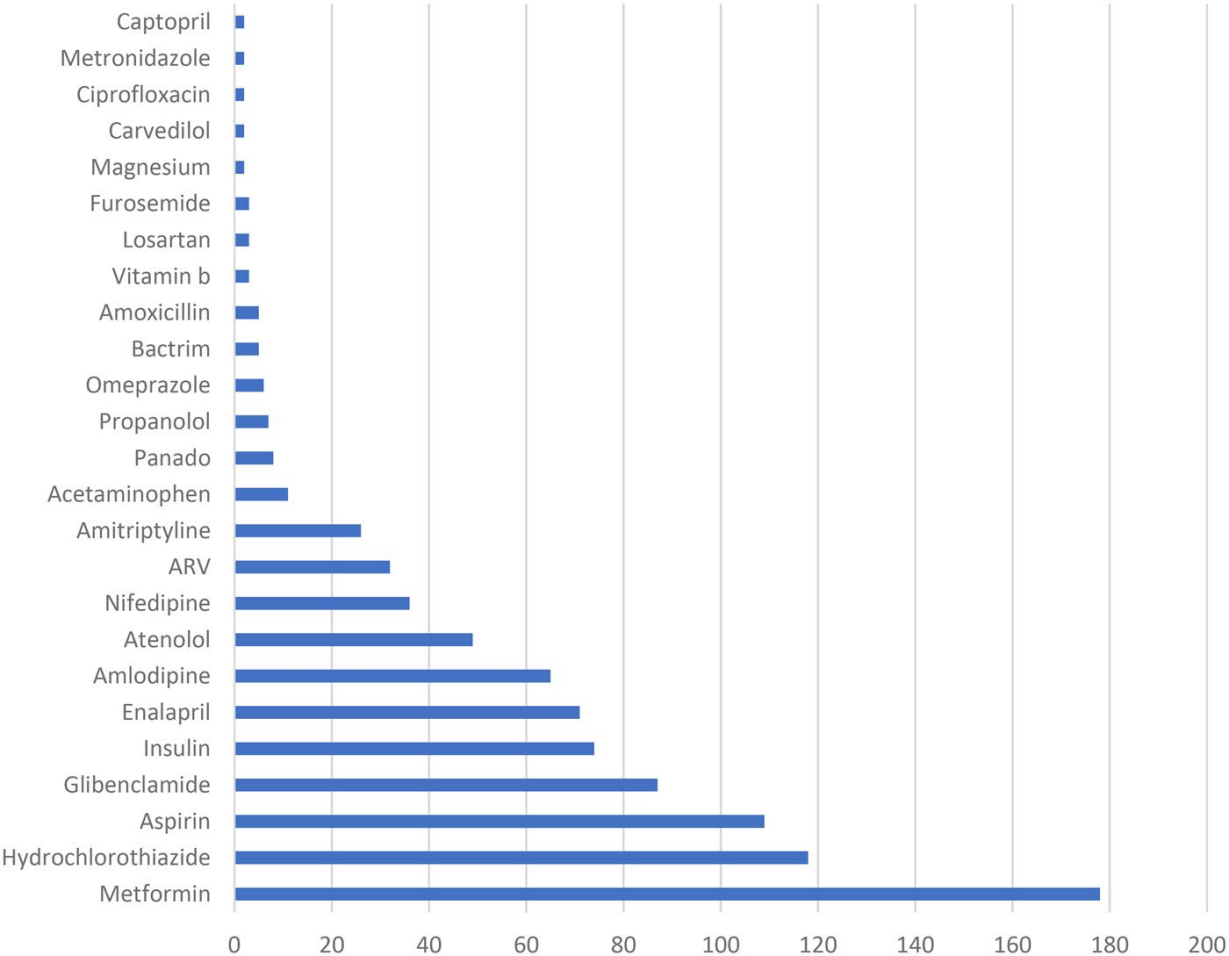
Most commonly reported pharmaceuticals by study participants. ARV, anti-retroviral.

Patients reported using a total of 96 different herbal products. The most commonly reported herbal products are listed in Figure 1, and a complete list of herbal products mentioned more than once is included in Supplemental File 3. All data are presented in Supplemental File 2. The most commonly reported pharmaceutical drugs are listed in Figure 2.

Patients were taking both pharmaceutical drugs and herbal products, with the most common herbal products being garlic, ginger, lemon, moringa, okra, and mango (Tables 3 and 4). A limited number (163) of participants provided comments about perceived positive or negative effects of the herbal products or pharmaceutical drugs they were taking. The comments provided, e.g., ‘More strength and the blood pressure levels are stable’ and ‘Quick digestion’ are captured in the full data set available in Supplemental File 2. An insufficient quantity and depth of responses were collected, precluding qualitative coding and analysis of themes.

**Table 3.** Most common herbal products used by participants (*n* = 301) taking the top three ant-hypertensive or anti- hyperglycemic pharmaceutical drugs.

Most common herbal products for patients taking hydrochlorothiazide, enalapril, or amlodipine (hypertension treatment)		Most common herbal products for patients taking metformin, insulin, or glibenclamide (diabetes treatment)	
Garlic	27.9% (84)	Ginger	27.2% (82)
Ginger	27.2% (82)	Garlic	21.9% (66)
Lemon	10.6% (32)	Okra	19.6% (59)
Moringa	7.3% (22)	Mango	14.3% (43)
Okra	6.0% (18)	Moringa	12.0% (36)

### 3.2. Herbal-drug interaction potential

**Figure 3.**
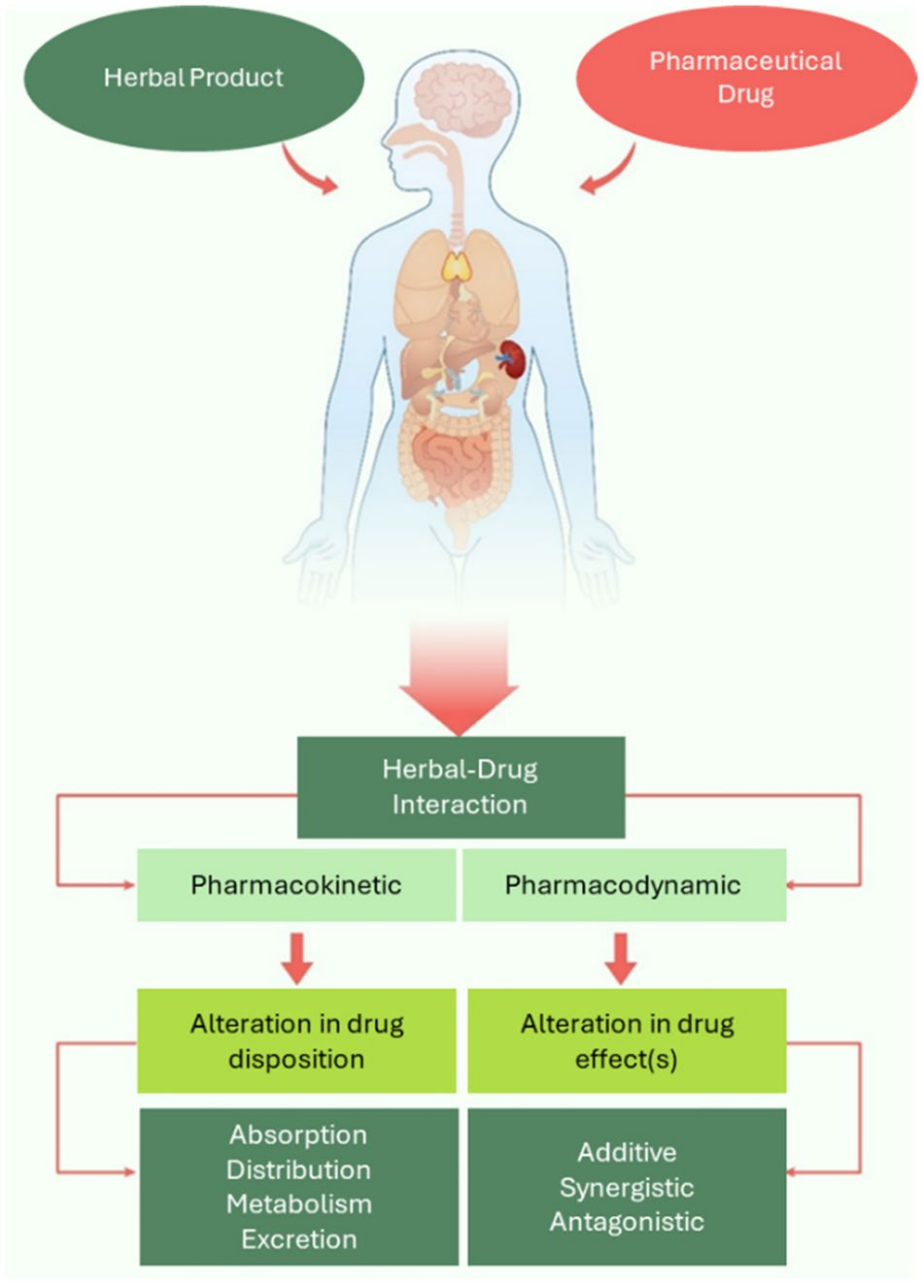
Overview of the mechanisms of herbal-drug interactions. Adapted from (Chatterjee et al. 2022).

This study examined the concurrent use of herbal products and pharmaceutical drugs in patients undergoing treatment for diabetes and/or hypertension at a large hospital in Malawi. As shown in Figure 1, preparations made with ginger, garlic, okra, lemon, moringa, mango, guava, Teras juice (a proprietary herbal mixture), hibiscus, and kigelia were the most frequently used. These observations are consistent with a previous publication, in which patients reported aloe, moringa, ginger, and garlic as the most frequently used medicinal plants by patients of the QECH in Blantyre (Mponda et al. 2024). The potential for HDIs associated with the most commonly reported herbal products (garlic, ginger, lemon, okra, mango, and moringa) are discussed. The concomitant pharmaceutical drugs and their key ADME properties are presented (Table 5). We evaluated the published literature for the PK and PD of herbal products in combination with the pharmaceuticals that patients reported taking (Figure 3). Regarding PK, the effects of the herbal products and their key constituents on drug metabolizing enzymes and transporters were reviewed. Effects on drug absorption, distribution, and excretion were evaluated if available.

While most herbal products have been shown to potentially interact with pharmaceutical drugs, and some have even been shown to impact the metabolism of such drugs, clinically relevant HDIs have been noted only for a handful of herbal products (Gurley et al. 2012; Sprouse and van Breemen 2016; Gurley 2025). Therefore, any report of a possible interaction does not mean that the medicinal plant is actually affecting the therapeutic outcome.

Regarding PD, studies were reviewed that evaluated PD changes when these herbal products were taken concurrently with the anti-hyperglycemics metformin, insulin, and/or glibenclamide, as well as anti-hypertensives hydrochlorothiazide, enalapril, or amlodipine. Finally, we discuss potential HDIs or toxicities associated with the less frequently reported herbal products, namely aristolochia and tamarind. These case examples were selected based on prior knowledge of their pharmacological mechanisms or potential toxicities. We have summarized the literature but did not assess study quality, as the literature includes a mix of *in vitro*, animal, and human data, as well as studies on whole plant extracts versus isolated constituents. Accordingly, our goal is to provide an overview of the available research and highlight gaps for future investigation.

#### 3.2.1. Garlic (*Allium sativum* L.)

Garlic is one of the most extensively studied and widely consumed herbal products worldwide (Furhad and Bokhari 2024). Used for centuries in traditional medicine, garlic has been associated with numerous purported health benefits, ranging from cardiovascular support to antimicrobial activity. While human studies show mixed results regarding garlic’s ability to lower cholesterol or blood pressure, most systematic reviews suggest some health benefit from garlic supplementation. Beyond these medicinal roles, garlic is a staple culinary ingredient. The bioactive constituents in garlic, including allicin, S-allyl-L-cysteine, and related organosulfur compounds, are believed to be responsible for these health effects. Such constituents may also influence the metabolism and activity of pharmaceutical drugs, raising concerns for HDIs when garlic is used concurrently with pharmaceutical drugs. In the current study, 120 patients reported taking garlic in some form for medicinal purposes.

Several studies have examined the impact of garlic preparations on drug metabolizing enzymes and transporters using both *in vitro* and human clinical data. *In vitro* studies have shown that garlic constituents inhibit CYP enzymes, including CYP2C9, CYP3A4, and CYP2E1 (Hajda et al. 2010; Ho et al. 2010). These same studies reported induction of transport proteins such as P-gp and organic anion transporting polypeptide (Oatp) 1a5. However, Oatp1a5 is a rat isoform of the OATP/Oatp family, and its regulation may not reflect human transporter responses. Moreover, the inducibility of OATPs in humans remains controversial, with limited and inconsistent evidence.

In contrast to *in vitro* findings, clinical studies have provided more direct insight into potential *in vivo* effects. For example, studies in healthy adults consuming garlic oil showed significant reductions in CYP2E1 activity in both young and older adults (Gurley et al. 2002, 2005). Such enzyme modulation may alter the pharmacokinetics of drugs commonly used to treat diabetes and hypertension. Nonetheless, Gurley et al. (2012) concluded that most CYP2E1 substrate drugs (e.g., acetaminophen, chlorzoxazone) have relatively wide therapeutic indices, suggesting that these interactions are unlikely to pose substantial clinical concern.

In diabetic animal models, garlic enhanced the therapeutic effects of the anti-hyperglycemic drugs metformin and glibenclamide, resulting in improved heart protection and greater reductions in blood glucose levels relative to control animals (Poonam et al. 2013; Asdaq et al. 2021). Similar effects have been reported with the anti-hypertensive drug enalapril, where co-administration with garlic in rodents led to greater decreases in blood pressure and triglycerides compared to either treatment alone (Elkayam et al. 2001). While these findings suggest that garlic may complement certain pharmaceutical drug effects under specific conditions, the underlying mechanisms were not clearly established and may reflect overlapping biological activity rather than changes in drug exposure.

Other studies have identified potential pharmacokinetic interactions involving garlic. For example, *in vitro* data suggest that garlic can induce human P-gp, which may affect the absorption and plasma concentrations of certain pharmaceutical drugs. These data may help explain the modest reduction in saquinavir levels observed in human volunteers after three weeks of consuming a standardized garlic extract (Hajda et al. 2010). Documented interactions between garlic and pharmaceutical drugs used to treat HIV, including saquinavir, may be of concern (Piscitelli et al. 2002; Gardner et al. 2007; NCCIH 2020). However, the overall risk of clinically significant HDIs involving garlic remains low based on current evidence.

These findings underscore the need for more clinical research to determine the relevance of garlic’s pharmacological effects at dietary versus herbal product doses, as well as its potential to influence the pharmacokinetics and pharmacodynamics of concurrently administered pharmaceutical drugs.

#### 3.2.2. Ginger (*Zingiber officinale* roscoe)

Ginger, a plant native to Asia, has been used in traditional Chinese medicine for over 2,500 years and remains a common remedy for digestive issues, particularly nausea (NCCIH 2025). Derived from the rhizome, ginger is consumed both as a culinary spice and as a dietary supplement and is generally regarded as safe. Its medicinal properties have been investigated in a variety of contexts, including metabolic and cardiovascular conditions (Ghayur and Gilani 2005; Ebrahimzadeh et al. 2022). While its popularity persists, the clinical evidence regarding its role in therapeutic applications beyond nausea is limited. For diabetes or hypertension treatment in the current study, 133 patients reported taking ginger.

Scientific studies suggest that ginger may influence drug metabolism and disposition. It has been shown to inhibit several key drug metabolizing enzymes and transporters *in vitro*, including CYP3A4, CYP1A2, CYP2C8, and P-gp (Zhang and Lim 2008; Yu et al. 2011; Mukkavilli et al. 2014). Further, ginger has been reported to reduce the oral bioavailability of cyclosporine in animal models (Chiang et al. 2006). While these actions could affect the absorption and bioavailability of co-administered drugs, there is no clinical evidence to support such interactions. A concern with the concomitant use of ginger with blood-thinning medications (e.g., aspirin, clopidogrel, warfarin) or prior to surgery is its potential impact on platelet aggregation. A systematic review of clinical and observational studies assessing the effects of ginger intake on platelet aggregation reported that of eight clinical studies, four reported that ginger increased aggregation time, while the other four studies did not find any effects (Marx et al. 2015). Similarly, the two observational studies reported mixed findings.

Ginger has also been evaluated for its effects on metabolic and vascular outcomes, which may be relevant to potential pharmacodynamic interactions when used with pharmaceutical drugs. For example, a clinical study in patients newly diagnosed with type II diabetes reported that co-administration of ginger with metformin was associated with reductions in body weight, fasting glucose, and cholesterol (El Gayar et al. 2019). In diabetic rodent models, ginger combined with glibenclamide showed additive effects in reducing oxidative stress and improving glycemic control (Ahmadi et al. 2013; Alshathly 2019). Regarding blood pressure, findings from animal studies and a limited number of human trials suggest that higher doses of ginger may contribute to modest reductions, particularly among younger adults, as noted in a meta-analysis (Hasani et al. 2019; Alam et al. 2021). While these data suggest that ginger may influence the same physiological targets as certain pharmaceutical drugs, the clinical evidence is still emerging, and further research is needed to clarify whether co-use enhances therapeutic effects or introduces risks of HDIs.

#### 3.2.3. Lemon (*citrus × Limon* (L.) osbeck)

Lemon is a globally cultivated fruit that originated in Asia and has long been used in both culinary and traditional medicinal contexts. Known for having high vitamin C content, lemon is commonly consumed to support immune function, enhance skin health, and improve iron absorption. Lemon is also frequently used as a digestive aid and is popular as a flavoring agent in both food and beverage preparations (WFO 2023b). While lemon is widely recognized as a safe and health-promoting food, its potential interactions with medications are less well characterized compared to other botanicals. Fifty participants in the current study reported taking lemon.

Emerging evidence suggests that lemon and its bioactive compounds, particularly limonin, may affect drug metabolism and transport. *In vitro* studies indicate that lemon can inhibit CYP3A4 (Baltes et al. 2001; Petric et al. 2020; Iampanichakul et al. 2023). Limonin has also been shown to inhibit CYP1A2, CYP2C8, CYP2C9, CYP2C19, and CYP2D6, as well as P-gp (Han et al. 2011), suggesting that co-administration with pharmaceuticals could theoretically alter their PK and efficacy. Despite these findings, clinical evidence remains sparse. A few animal studies point to potential anti-hyperglycemic effects; for example, lemon leaf extract lowered blood glucose in hyperglycemic mice comparable to glibenclamide (Kumar and Rajput 2022), and naringenin, a flavanone found in citrus fruits, altered the distribution of metformin in diabetic rats (Mato Mofo et al. 2020). However, interactions between lemon and common antihypertensive medications such as enalapril or amlodipine have not been studied in detail. Because lemon is broadly consumed and generally regarded as safe in dietary amounts, its impact on drug metabolism is not likely to be clinically relevant. However, its use at higher doses or in supplemental forms warrants further investigation to assess the potential for HDIs.

#### 3.2.4. Okra (*Abelmoschus esculentus* (L.) Moench)

Okra is a flowering plant known for its edible seed pods and is widely cultivated across Africa, the Middle East, South Asia, and the southern United States. Traditionally used as both a food and folk remedy, okra is reported to support digestive health and help regulate blood sugar levels, which has led to its growing popularity in traditional approaches to managing diabetes (WFO 2023a). In the current study, 59 participants reported using okra, including those concurrently taking common antihypertensive or antidiabetic medications.

Although little is currently known about how okra influences drug metabolizing enzymes, existing evidence suggests it may affect object drug PK and efficacy. For example, animal studies have indicated that okra extract can reduce the absorption and effectiveness of metformin (Khatun et al. 2011; Haque et al. 2022).

Clinical and preclinical studies suggest that okra may improve lipid profiles and blood glucose levels in patients with diabetes, with some effects comparable to glibenclamide (Amadi et al. 2021; Mokgalaboni et al. 2023; Tavakolizadeh et al. 2023). Okra has also demonstrated antihypertensive and cholesterol-lowering effects in rat models, occasionally outperforming standard treatments like enalapril (Mondal et al. 2019). Despite these promising findings, the evidence also indicates a potential risk of pharmacodynamic HDIs, particularly with metformin, where reduced drug efficacy could compromise glucose control. Nevertheless, the glucose-lowering effects of okra are modest at best, and hence unlikely to be clinically relevant.

#### 3.2.5. Mango (*Mangifera indica*)

Mango is a widely consumed fruit that originated in Southeast Asia and is now cultivated globally. With hundreds of cultivars differing in appearance and flavor, mango is a popular dietary fruit. However, it is mainly the leaf that is recognized for its potential health benefits (Table 4). Rich in fiber, vitamin C, and vitamin A, mango fruit is often promoted for its digestive and nutritional properties (WFO 2023c). In the current study, 46 participants reported using mango leaf, including 43 individuals who were also taking antidiabetic medications such as metformin, insulin, or glibenclamide.

**Table 4.** Most common herbal products reported from the survey plus other herbal products selected by authors based on potential herbal-drug interactions or toxicity.

Herbal name	Latin Name	Common uses people report taking the herbal product	Form(s) taken based on survey
Ginger	*Zingiber officinale* Roscoe	Digestive aid, anti-inflammatory	Parts - root Form - raw, powder, solvent extract, tea
Garlic	*Allium sativum* L.	Antibacterial, heart health	Parts - bulb Form - raw, powder, solvent extract
Lemon	*Citrus* × *limon* (L.) Osbeck	Vitamin C source, detoxifying	Parts - fruit Form - juice, solvent extract
Moringa	*Moringa oleifera* Lam.	Nutritional supplement, anti-diabetic	Parts - leaves Form - powder, solvent extract
Okra	*Abelmoschus esculentus* (L.) Moench	Dietary fiber source	Parts - fruit Form - solvent extract
Mango	*Mangifera indica* L.	Nutritional, anti-inflammatory	Parts - leaves Form - solvent extract, leaves
Tamarind	*Tamarindus indica* L.	Digestive, sore throat remedy	Parts - fruit Form - powder, juice, solvent extract
Aristolochia	*Aristolochia* spp. (L.)	Pain relief, anti-inflammatory, wound healing, digestive issues	Not specified

**Table 5.** Pharmaceutical drugs commonly used for treatment of hypertension and diabetes and key.

Pharmaceutical Drug	Drug Class	Mechanism of Action	Absorption	Distribution	Metabolism	Excretion	Interactions List (not exhaustive)
Amlodipine	Dihydropyridine calcium channel blocker	Inhibits calcium influx in muscle, reducing vascular resistance and lowering blood pressure	High but slow absorption from GI tract; no impact of food on absorption	Large Vd; distribution to vascular smooth muscle; high plasma protein binding	Predominantly metabolism by hepatic CYP3A	Excreted into urine as metabolite; long half life	clarithromycin, cyclosporine, diltiazem, itraconazole, simvastatin, tacrolimus
Enalapril	Angiotensin-converting enzyme (ACE) inhibitor	ACE inhibition lowers angiotensin II production & plasma levels, increases plasma renin activity, & decreases aldosterone secretion. Reduced angiotensin II levels lead to peripheral vasodilation & decreased vascular resistance, lowering blood pressure	60% bioavailable orally; no impact of food on absorption	Large Vd; distributed to liver, metabolite distributed to kidneys, heart, and vascular endothelium; moderate plasma binding	A prodrug; requires hepatic metabolism for activity (by hepatic esterase)	Excreted into urine and feces	cardiovascular meds, anti-hyperglycemics, specific diuretics, NSAIDs, steroids, potassium supplements, alcohol
Glibenclamide	Second generation sulfonylurea	Lowers blood glucose by stimulating insulin release from pancreatic beta cells; binds to ATP-sensitive potassium channels, causing closure, depolarization, and calcium influx, which triggers insulin secretion	95% bioavailable orally; well-absorbed from GI tract; lower when taken with a meal	99% bound to plasma proteins; moderate Vd with even distribution between blood and tissues due to high protein binding; distributed throughout the body, including pancreas	Metabolized mainly by CYP3A, followed by CYP2C9 and CYP2C19	Excreted as metabolite, 50% into the urine and 50% into the feces	bosentan; aminolevulinic acid, eluxadoline, ethanol, fluvastatin, ivacaftor, lumacaftor/ivacaftor, methyl aminolevulinate
Hydrochlorothiazide	Thiazide diuretic	Transport into distal convoluted tubule cells *via* OAT1, OAT2, OAT4. Transport to tubule lumen by MRP4; Inhibits SLC12A3, reducing water reabsorption	65–75% bioavailable orally; 10% lower when taken with a meal	Variable Vd (moderate - high)	Not metabolized	Excreted into urine as parent	cholestyramine, colestipol, cardiovascular meds (including digoxin, dofetilide, anti-hypertensives), anti-hyperglycemics, muscle relaxants, diuretics, steroids (prednisone, cortisone)
Insulin	Natural hormone	Regulates glucose metabolism by facilitating glucose & amino acid uptake into muscle & fat, enhancing glycogen, fatty acids, & protein synthesis, & suppressing liver gluconeogenesis. Activates the insulin receptor’s tyrosine kinase, which phosphorylates substrates & triggers pathways such as PI3 kinase & Akt, essential for regulating GLUT4 & protein kinase C in metabolism	Absorbed into the bloodstream from subcutaneous injections. Absorption rate varies with injection site and formulation and is ineffective when taken orally.	Distributes rapidly to body tissues, especially muscle and fat, but not to the brain. Has a short half-life in the bloodstream	Primarily metabolized in liver and kidneys by IDE and cathepsin B	Eliminated *via* metabolism in liver and kidneys	alcohol, antibiotics (ciprofloxacin, levofloxacin, sulfamethoxazole/ trimethoprim), cardiovascular meds (benazepril, enalapril, lisinopril, losartan, valsartan, beta blockers like atenolol, metoprolol, propranolol), mental health meds (fluoxetine, olanzapine), diuretics (HCTZ), hormones (estrogen, progestin, testosterone, thyroid hormones), anti-hyperglycemics, steroids (prednisone, cortisone), anti-hypertensives (clonidine, guanethidine, reserpine)
Metformin	Biguanide antihyperglycemic	Activates AMPK, reducing hepatic gluconeogenesis by inhibiting PEPCK & G6Pase, & blocks mitochondrial complex I, boosting AMP levels and AMPK activity. Enhances muscle glucose uptake *via* GLUT4 & slightly decreases intestinal glucose absorption, lowering blood glucose without significant hypoglycemia	50–60% orally bioavailable (*via* tablet) in fasting state; food reduces absorption; mainly absorbed in the intestines	Large Vd; distributed to liver, intestines and kidney; low plasma binding	Not metabolized; drug-drug interactions minimal	Excreted into urine as parent	acetazolamide, alcohol, antivirals, cardiovascular medicines, cimetidine, digoxin, diuretics, hormones, phenothiazines, steroids, stimulants, thyroid medicines, miscellaneous medicines (including dichlorphenamide, glycopyrrolate, isoniazid, lamotrigine, memantine, methazolamide, metoclopramide, midodrine, niacin, phenytoin, ranolazine, topiramate, trospium, vandetanib, zonisamide)

ADME information: AMP: Adenosine monophosphate; AMPK: AMP-activated protein kinase; ATP: Adenosine triphosphate; CYP: Cytochrome P450 (e.g., CYP3A, CYP2C9); G6Pase: Glucose-6-phosphatase; GI: Gastrointestinal; GLUT4: Glucose transporter type 4; HCTZ: Hydrochlorothiazide; IDE: Insulin-degrading enzyme; MRP4: Multidrug resistance-associated protein 4; NSAIDs: Nonsteroidal anti-inflammatory drugs; OAT: Organic anion transporter (e.g., OAT1, OAT2, OAT4); PEPCK: Phosphoenolpyruvate carboxykinase; PI3 kinase: Phosphoinositide 3-kinase; SLC12A3: Solute carrier family 12 member 3; Vd: Volume of distribution.

Scientific investigations suggest that mango fruit may influence the CYPs and drug transporters. Some studies have shown that mango inhibits rodent Cyp1a1, Cyp1a2, Cyp3a1, Cyp2c6, and Cyp2e1, as well as P-gp (Adisa et al. 2015; Showande et al. 2019). Mango has also been reported as a moderate inhibitor of human CYPs, including CYP2C8, CYP2B6, CYP2D6, and CYP2C9 (Rodríguez-Fragoso et al. 2011). These interactions suggest a potential for mango fruit to alter the PK of drugs that are substrates for these enzyme, although the clinical significance remains to be fully elucidated (Rodeiro et al. 2013; Sekar et al. 2020). No studies assessing the risk of potential HDIs of mango leaves could be retrieved for this review.

Regarding therapeutic effects, preclinical studies have demonstrated promising anti-hyperglycemic properties associated with various mango plant parts. Mango leaf and peel extracts have been shown to lower blood glucose levels in rats, with effects comparable to metformin (Gondi and Prasada Rao 2015; Aqyun et al. 2019). *In vitro* studies highlight mango seeds as particularly active in enhancing glucose uptake and inhibiting α-amylase, an enzyme involved in carbohydrate digestion (Ahmad et al. 2023). Additional work suggests that mango kernel flour and mango leaves may influence the PD of metformin by enhancing its glucose-lowering effects (Irondi et al. 2016; Lal et al. 2023). However, because different studies focus on different mango parts (e.g., leaves, seeds, kernels, or fruit pulp), the findings are not directly comparable, and further research is needed to clarify how mango leaf intake may influence medication efficacy or safety in patients with diabetes.

#### 3.2.6. Moringa (*Moringa oleifera* Lam.)

Moringa (*Moringa oleifera*), also known as the drumstick or horseradish tree, is a nutrient-rich plant native to the Himalayan region and now cultivated widely across tropical and subtropical climates. Various parts of the plant (e.g., leaves, pods, and seeds) are consumed for both food and traditional medicinal purposes, with the leaf being the most commonly used component in herbal products (Nova et al. 2020). Moringa is often touted for its antioxidant, anti-inflammatory, and anti-hyperglycemic properties, making it a popular choice among individuals seeking natural approaches to managing chronic conditions. In the current study, 46 participants reported using moringa.

Moringa has been shown to inhibit several drug metabolizing enzymes. *In vitro* studies using methanolic, ethanol, and aqueous extracts of moringa leaves indicated inhibitory effects on CYP3A4, CYP1A2, CYP2D6, and CYP2E1 (Monera et al. 2008; Taesotikul et al. 2010; Ahmmed et al. 2015; Fantoukh et al. 2019). These effects suggest that moringa may alter the metabolism of co-administered pharmaceuticals, potentially affecting their PK or PD. This concern is supported by clinical and animal studies showing altered PK of certain drugs; for instance, co-administration of moringa with the antimalarial drug amodiaquine significantly reduced the maximum plasma concentration of amodiaquine in human adults, and increased plasma concentrations of rifampicin in mice (Olawoye et al. 2018; Pal et al. 2018). On the other hand, a study in patients with HIV looking at a possible interaction between the antiretroviral drug nevirapine and moringa leaf powder did not show any alterations in nevirapine plasma concentrations. Because amodiaquine is metabolized by CYP2C8, contrarily to nevirapine, which is metabolized *via* CYP3A4 and CYP2B6, this may point to differences in the affinity of moringa leaf constituents to various CYPs (Monera-Penduka et al. 2017).

Although only a few controlled clinical studies exist, results generally support moringa’s potential anti-hyperglycemic activity. Because several participants in the current study reported taking both moringa and metformin, this combination may increase the risk of hypoglycemia, and clinicians should be attentive to such concurrent use. As with many herbal products, further research is needed to better understand the HDI potential and safety profile of moringa, especially in populations managing chronic diseases with pharmaceutical drugs.

#### 3.2.7. Tamarind (Tamarindus indica L.)

Tamarind is a tropical tree believed to have originated in West Africa but is now widely cultivated across Asia, particularly in India. Traditionally, tamarind has been used for a variety of medicinal purposes, including as a laxative, digestive aid, and treatment for ailments such as wound infections, abdominal discomfort, parasitic infections, fever, and malaria. The pulp, rich in organic acids and polyphenols, is commonly consumed in food and beverages, often appreciated for its tangy flavor and digestive benefits. Although tamarind was not one of the most commonly used herbal products (7 participants reported taking tamarind), the authors selected this one of interest for potential HDIs.

Tamarind has demonstrated the potential to alter the pharmacokinetics of some drugs. For example, tamarind been shown to inhibit CYP3A4 in a dose-dependent manner in human cells, indicating that it may interfere with the metabolism of drugs that are substrates for this enzyme (Roy and Lakshmi 2018). Additionally, in a clinical human study, tamarind has been reported to increase the bioavailability of certain medications, such as aspirin, likely by affecting gastrointestinal absorption (Mustapha et al. 1996).

An extract of tamarind fruit pulp has been shown to potentially affect drug pharmacological effects *in vitro*. Its laxative properties may contribute to pharmacokinetic interactions by decreasing the intestinal transit time of pharmaceutical drugs. When combined with other herbal products with similar effects, there may be a risk of additive consequences such as electrolyte imbalance (e.g., hypokalemia), dehydration, or metabolic alkalosis. These findings suggest that caution should be taken when tamarind is used in conjunction with other medications, particularly those affecting hydration or electrolyte balance. However, studies on the risk of HDIs for tamarind fruit in humans are lacking, and hence the clinical relevance of the *in vitro* findings remains to be determined.

#### 3.2.8. Aristolochia spp. (L.)

*Aristolochia* spp. are a family of plants whose medicinal use raise significant safety concerns due to the presence of aristolochic acids, particularly aristolochic acid I and II. These compounds are well-documented for their genotoxic and nephrotoxic properties, and their oral use has been associated with serious health risks, including kidney failure, urothelial cancers, and even death (Grollman 2013; Boot et al. 2020). In the current study, four participants reported using herbal products believed to be from *Aristolochia* spp. Although the exact chemical composition of these preparations was not determined, the possibility of aristolochic acid content presents a major public health concern.

Aristolochic acid I has been identified as a substrate for breast cancer resistance protein (BCRP), a human ABC efflux transporter (Ma et al. 2015). This observation suggests the potential for interactions with other substrates or inhibitors of BCRP, such as the anticancer drugs methotrexate and irinotecan, or some cholesterol-lowering drugs like rosuvastatin, which could affect the PK of these pharmaceutical drugs. While the literature is sparse on detailed PD studies specific to *Aristolochia* spp. in combination with pharmaceutical drugs, the presence of the highly toxic compounds in *Aristolochia* spp. mandates extreme caution, particularly in populations concurrently taking pharmaceutical medications that may interact at shared transport or metabolic pathways. Due to the well-established toxicological profile of aristolochic acids, healthcare providers should strongly discourage the use of *Aristolochia*-containing products and prioritize patient education and monitoring if use is suspected.

### 3.3. Future research needs and limitations

This exploratory study highlights several areas where additional research is needed. Healthcare providers should routinely document patients’ use of herbal products, as such practices are often underreported. Although this study was conducted in Malawi, many of the herbal products identified are commonly used across Africa. This observation indicates that the results may be relevant beyond Malawi and support expanding similar surveys to other African regions and low- and middle-income countries where the concurrent use of herbal products and pharmaceutical drugs is widespread. Doing so would improve our understanding of HDIs on a broader scale and help inform globally relevant clinical and public health guidance.

Results also underscore the need for increased education for both healthcare providers and patients about the potential risks and benefits of combining herbal products with pharmaceutical drugs. Adapting and replicating this survey in diverse geographic and clinical settings could lead to safer, more informed use of herbal products alongside pharmaceutical drugs.

Several limitations of the current study must be acknowledged. First, the herbal products reported may not reflect what is actually used in Malawi due to variability in preparation methods (e.g., tea versus supplement) or lack of plant identification (the same vernacular name may be used for different species). Second, this study relied on patient self-reports, which introduce potential inaccuracies and limit data verification. Third, participants often used the terms ‘hypertension’ and ‘high blood pressure’ interchangeably, which may not accurately reflect a formal medical diagnosis and could introduce ambiguity when interpreting the clinical relevance of reported conditions or the appropriateness of herbal product use. Fourth, the use of convenience sampling limits the generalizability of the results. Fifth, many herbal products are complex mixtures containing hundreds of bioactive compounds with varying pharmacological properties. Without chemical characterization or analytical validation, drawing conclusions about specific interactions or health effects is difficult (Collins et al. 2020; Mitchell et al. 2022). Future studies should incorporate appropriate analytical techniques and representative sampling to improve the reliability and applicability of findings in this field.

Finally, although some participants provided comments about perceived effects of herbal products or pharmaceutical medicines, fewer than 10% responded and most comments were brief. These observations limited the ability to conduct a rigorous qualitative analysis. Nonetheless, we reported examples of these responses in the supplemental materials. Future studies specifically designed to include structured qualitative components will be needed to better capture patient perspectives and real-world experiences.

## 4. Summary and conclusions

Results from this exploratory study serve as a foundation for future hypothesis-driven research studies. Investigating HDIs, especially those involving understudied herbal products, remains a key priority, both globally and in Malawi. Further work is also needed to characterize the chemical composition of these products and evaluate their safety and efficacy in real-world use. The complexity of herbal products, variation in usage practices, and lack of standardization render challenges in assessing their true health impact. These challenges are not unique to Malawi and are likely reflected in other parts of the world. Additionally, while we focused on diabetes and hypertension, this methodology could be applied to other diseases or areas in the world. This study contributes important baseline data and identifies critical gaps for further investigation to support safer integration of herbal products and pharmaceutical drug treatments.

## Supplementary Material

Supplemental 3 Herbal Products and Pharmaceutical Medicines List Supplemental.xlsx

Suppl 1 Herb Drug Interaction Survey.docx

Supplemental 2 HDI Project Full Dataset Supplemental.xlsx

## Data Availability

Data are available open access as part of the supplementary files.

## CRediT roles

- Kumbukani Nyirenda: Conceptualization, Methodology, Formal Analysis, Investigation, Data Curation, Writing – Original Draft, Visualization, Supervision, Funding Acquisition, Writing – Review & Editing
- John Mponda: Methodology, Investigation, Data Curation, Writing – Original Draft, Writing – Review & Editing
- Ibrahim Chikowe: Methodology, Investigation, Data Curation, Writing – Original Draft, Writing – Review & Editing
- Esther Kawonga: Investigation, Data Curation, Writing – Review & Editing
- Nellie Twatasha Phiri: Investigation, Data Curation, Writing – Review & Editing
- Mervis Msukwa: Investigation, Data Curation, Writing – Review & Editing
- Chimota Phiri: Investigation, Data Curation, Writing – Review & Editing
- Amy Roe: Formal Analysis, Writing – Original Draft, Writing – Review & Editing
- Mary F. Paine: Formal Analysis, Writing – Original Draft, Writing – Review & Editing
- Bill Gurley: Formal Analysis, Writing – Original Draft, Writing – Review & Editing
- Hellen Oketch-Rabah: Formal Analysis, Writing – Original Draft, Writing – Review & Editing
- Stefan Gafner: Data Curation, Writing – Original Draft, Writing – Review & Editing
- Julie Krzykwa: Software, Formal Analysis, Resources, Writing – Review & Editing
- Constance A. Mitchell: Software, Formal Analysis, Resources, Data Curation, Writing – Original Draft, Visualization, Project Administration, Writing – Review & Editing
- Michelle Embry: Conceptualization, Software, Formal Analysis, Resources, Data Curation, Writing – Original Draft, Visualization, Project Administration, Funding Acquisition, Writing – Review & Editing
- Syril Pettit: Conceptualization, Resources, Writing – Original Draft, Project Administration, Funding Acquisition, Writing – Review & Editing
- Dallas J. Smith: Conceptualization, Methodology, Investigation, Data Curation, Supervision, Funding Acquisition, Writing – Review & Editing.
