# Supplementary material for: An exploratory evaluation of the interaction risk between herbal products and pharmaceutical medicines used concurrently for disease management in Blantyre, Malawi
Source: Pharm Biol. 2025 Nov 19;63(1):877–95. doi: 10.1080/13880209.2025.2586351 (PMC12632214; doi:10.1080/13880209.2025.2586351)
Supplement: Suppl 1 Herb Drug Interaction Survey.docx [file IPHB_A_2586351_SM8436.docx]

**QUESTIONNAIRE: PATIENTS ATTENDING HYPERTENSION AND DIABETES CLINIC AT QUEEN ELIZABETH CENTRAL HOSPITAL**

**SERIAL NO.**

**NAME OF CLINIC: …………………………….**

**DATE: …………………………….**

***Objective:*** *To identify conventional medicines and herbal medicine concurrently used for the management of NCDs in NCD clinics in Malawi*

**Sociodemographic information**

1. Residence**:** Urban Peri urban Rural/Village Other: ………….…
2. Sex. Male Female
3. Age Years
4. Religion: Christian Muslim Other ……………………
5. Marital Status: Married Divorced Widowed Unmarried
6. Educational Status: Primary School Secondary School College and above
7. Employment status: Full time Part time Unemployed Business

Other………………………

**Clinical information on high blood pressure (hypertension)**

1. Do you have high blood pressure (hypertension)? Yes No
2. What are your current blood pressure readings? (check in health passport): ……………mmHg
3. When were you first diagnosed with high blood pressure (hypertension)?

less than 1 year ago 1-2 years ago more than 2 years ago

1. Are you taking any conventional medicines (for example, hydrochlorthiazide, bendrofluazide, amlodipine, nifedipine, etc.) for high blood pressure? Yes No
2. If Yes, please note which conventional medication(s) you are taking for high blood pressure, what dose, and how often they are taken:

| Conventional Medication Name | Dosage | Frequency |
| --- | --- | --- |
|  |  |  |
|  |  |  |
|  |  |  |
|  |  |  |

1. Does taking your conventional medication for high blood pressure make you feel badly? How? Describe?

| Conventional Medication | Bad effects | Description |
| --- | --- | --- |
|  |  |  |
|  |  |  |
|  |  |  |
|  |  |  |

1. Does taking your conventional medication for high blood pressure make you feel well/different?

| Conventional Medication | Good effects | Description |
| --- | --- | --- |
|  |  |  |
|  |  |  |
|  |  |  |
|  |  |  |

1. Are you prescribed or taking any other conventional medications? Yes No
2. If Yes, please note which additional conventional medication(s) you are taking, what dose, how often they are taken, and what you are using the medication to treat:

| Conventional Medication | Dose | Frequency | Indication |
| --- | --- | --- | --- |
|  |  |  |  |
|  |  |  |  |
|  |  |  |  |
|  |  |  |  |

1. Does taking your other conventional make you feel badly? How? Describe?

| Conventional Medication | Bad effects | Description |
| --- | --- | --- |
|  |  |  |
|  |  |  |
|  |  |  |
|  |  |  |

1. Does taking your other conventional medication make you feel well/different?

| Conventional Medication | Good effects | Description |
| --- | --- | --- |
|  |  |  |
|  |  |  |
|  |  |  |
|  |  |  |

**Clinical information on diabetes**

1. Are you diabetic? Yes No
2. What is your current sugar level measurement? (check in health passport) …………………….
3. When were you first diagnosed with diabetes?

less than 1 year ago 1-2 years ago more than 2 years ago

1. Are you taking any conventional medicines for your diabetes (for example, metformin)?

Yes No

1. If Yes, please note which conventional medication(s) you are taking for diabetes, what dose, and how often they are taken:

| Conventional Medication Name | Dosage | Frequency |
| --- | --- | --- |
|  |  |  |
|  |  |  |
|  |  |  |
|  |  |  |

1. Does taking your conventional medication for diabetes make you feel badly? How? Describe?

| Conventional Medication | Bad effects | Description |
| --- | --- | --- |
|  |  |  |
|  |  |  |
|  |  |  |
|  |  |  |

1. Does taking your conventional medication for diabetes make you feel well/different?

| Conventional Medication | Good effects | Description |
| --- | --- | --- |
|  |  |  |
|  |  |  |
|  |  |  |
|  |  |  |

1. Are you prescribed or taking any other conventional medications? Yes No
2. If Yes, please note which additional conventional medication(s) you are taking, what dose, how often they are taken, and what the medication is being used to treat:

| Conventional Medication | Dose | Frequency | Indication |
| --- | --- | --- | --- |
|  |  |  |  |
|  |  |  |  |
|  |  |  |  |
|  |  |  |  |

1. Does taking your other conventional make you feel badly? How? Describe?

| Conventional Medication | Bad effects | Description |
| --- | --- | --- |
|  |  |  |
|  |  |  |
|  |  |  |
|  |  |  |

1. Does taking your other conventional medication make you feel well/different?

| Conventional Medication | Good effects | Description |
| --- | --- | --- |
|  |  |  |
|  |  |  |
|  |  |  |
|  |  |  |

**Use of herbal medicinal products for high blood pressure (hypertension)**

1. Have you ever taken traditional medicines or herbal products for your high blood pressure (hypertension)? Yes No
2. Which products/medicinal plants do you take to treat your high blood pressure (hypertension) and what part of the plant is used?

| Name of Medicinal Plant/Product | Part of plant used |
| --- | --- |
|  |  |
|  |  |
|  |  |

1. What type(s) of traditional medicine or herbal products are used (for example, extract, powder, tincture), in what dose, and how often?

| Herbal Medicine Dosage Form | Dose | Frequency |
| --- | --- | --- |
|  |  |  |
|  |  |  |
|  |  |  |

1. How long have you been using traditional medicine or herbal products to treat your high blood pressure (hypertension)?

less than 1 year 1-2 years more than 2 years

1. What is the source of the traditional medicine or herbal products you are taking to treat your high blood pressure (hypertension)?

Pharmacy Herbalist clinic Market vendor Friends/family Garden/wild Other……………………………………….

1. Does taking your herbal medicine for high blood pressure make you feel badly? How? Describe?

| Conventional Medication | Bad effects | Description |
| --- | --- | --- |
|  |  |  |
|  |  |  |
|  |  |  |
|  |  |  |

1. Does taking your herbal medicine for high blood pressure make you feel well/different?

| Conventional Medication | Good effects | Description |
| --- | --- | --- |
|  |  |  |
|  |  |  |
|  |  |  |
|  |  |  |

1. Have you ever used traditional medicines or herbal products at the same time (same day) as the conventional medicines to treat high blood pressure (hypertension)? Yes No
2. Do you think that taking traditional medicines or herbal products to treat high blood pressure (hypertension) and conventional medicines to treat high blood pressure (hypertension) at the same time (same day) is: Helpful to treat my disease Not helpful to treat my disease

Harmful I don’t know Other …………………

_________________________________________________________________

**Use of herbal medicinal products for diabetes**

1. Have you ever taken traditional medicines or herbal products for your diabetes?

Yes No

1. Which products/medicinal plants do you take to treat your diabetes and what part of the plant is used?

| Name of Medicinal Plant/Product | Part of plant used |
| --- | --- |
|  |  |
|  |  |
|  |  |

1. What type(s) of traditional medicine or herbal products are used (for example, extract, powder, tincture), in what dose, and how often?

| Herbal Medicine Dosage Form | Dose | Frequency |
| --- | --- | --- |
|  |  |  |
|  |  |  |
|  |  |  |

1. How long have you been using traditional medicines or herbal products to treat your diabetes?

less than 1 year 1-2 years more than 2 years

1. What is the source of the traditional medicines or herbal products you are taking to treat your diabetes?

Pharmacy Herbalist clinic Market vendor Friends/family Garden/wild Other……………………………………….

1. Does taking your herbal medicine for diabetes make you feel badly? How? Describe?

| Conventional Medication | Bad effects | Description |
| --- | --- | --- |
|  |  |  |
|  |  |  |
|  |  |  |
|  |  |  |

1. Does taking your herbal medicine for diabetes make you feel well/different?

| Conventional Medication | Good effects | Description |
| --- | --- | --- |
|  |  |  |
|  |  |  |
|  |  |  |
|  |  |  |

1. Have you ever used traditional medicines or herbal products at the same time (same day) as the conventional medicines to treat diabetes? Yes No
2. Do you think that taking traditional medicines or herbal products to treat diabetes and conventional medicines to treat diabetes at the same time (same day) is:

Helpful to treat my disease Not helpful to treat my disease

Harmful I don’t know Other ……………………..

**Use of herbal medicinal products for other reasons**

1. Have you ever taken traditional medicines or herbal products for other conditions besides high blood pressure (hypertension) and/or diabetes?

Yes No

1. Which products/medicinal plants do you take to treat other conditions?

| Name of Medicinal Plant/Product | Part of plant used |
| --- | --- |
|  |  |
|  |  |
|  |  |

1. What type(s) of traditional medicine / herbal products are used (for example, extract, powder, tincture), in what dose, and how often?

| Herbal Medicine Dosage Form | Dose | Frequency |
| --- | --- | --- |
|  |  |  |
|  |  |  |
|  |  |  |

1. How long have you been using traditional medicines or herbal products to treat other conditions?

less than 1 year 1-2 years more than 2 years

1. What is the source of the herbal medicinal products you are taking to treat other conditions? Pharmacy Herbalist clinic Market vendor Friends/family Garden/wild Other……………………………………….
2. Does taking your herbal medicine for other conditions make you feel badly? How? Describe?

| Conventional Medication | Bad effects | Description |
| --- | --- | --- |
|  |  |  |
|  |  |  |
|  |  |  |
|  |  |  |

1. Does taking your herbal medicine for other conditions make you feel well/different?

| Conventional Medication | Good effects | Description |
| --- | --- | --- |
|  |  |  |
|  |  |  |
|  |  |  |
|  |  |  |

_________________________________________________________________

**END OF QUESTIONNAIRE**

**MAFUNSO : Kwa odwala nthenda ya kuthamanga kwa magazi ndi mtima kapena matenda shuga ku chipatala cha Queen Elizabeth Central.**

**SERIAL NO.**

**TSIKU: …………………………….**

**CHOLINGA *:*** Kufufuza za kuphatikiza pakamwedwe ka mankhwala akuchipatala ndi azisamba pa matenda othamanga magazi, a mtima ndi ashuga.

**Mbiri ya wodwala**

1. Malo okha**la:** Kutawuni Mmbali mwa tawuni Kumuzi Kwina:
2. Sex. Mwamuna Mkazi
3. Zaka
4. Chipembedzo: Chikhilisitu Chisilamu Chipembedzo china
5. Banja: Wokwatila Wosiyidwa pa banja Wamasiye Wosakwatira
6. Maphunziro: Pulayimale Sekondale Sukulu yaukachenjede
7. Ntchito: Ntchito yokhazikika Yosakhazikika Simuli pantchito Bizinesi

Ntchito zina ……………………………..

**Mbiri ya Nthenda Yothamanga Magazi ndi Mtima**

1. Mumadwala matenda othamanga magazi? Inde Ayi
2. Muyezo wa kathamangidwe ka magazi (check health passport) ……………………..mmHg
3. Ndiliti munapezeka ndi matendawo? <1year 1-2 years >2 years
4. Mukumwa mankhwala akuchipatala pa amatendawa? Eya Ayi
5. Ngati mukumwa, ndimankhwala anji (1) ………………………(ii) …………......

(iii) ……………………………….

1. Mankhwalawa mumaphatikiza ndimakhwala ena? Eya Ayi
2. Ngati Eya, mukuphatikiza ndimankhwala ati? (1) ……………………………. (ii) …………………………….
3. Ntchito ya mankhwala amenewa ndi chiyani (14) for? (i) ……………………………… (ii) …………………………….…………
4. Pali mavuto aliwonse amene abwela mthupi mwanu chifukwa cha mankhwala a BP? Eya Ayi
5. Ngati eya, ndi mavuto ati amene abwela mthupi mwanu chifukwa cha mankhwala a BP? (i) …………………………………….. (ii) ………..................................................

**Mbiri ya Nthenda Ya Diabetes**

1. Muli ndimatenda a shuga? Eya Ayi
2. Muyezo wa sugar mmagazi anu (Check health passport) ………………………………
3. Ndiliti munapezeka ndi matendawa? <1year 1-2 years >2 years
4. Mumamwa makhwala akuchipatala aliwonse a matendawa? Eya Ayi
5. Ngati Eya, mumamwa mankhwala anji amatendawa? (1) ………………………(ii) …………......

(iii) ……………………………….

1. Mankhwala amene mukumwawo mumaphatikiza ndi mankhwala ena? Eya Ayi
2. Ngati Eya, mukuphatikiza ndimankhwala ati? (1) ……………………………. (ii) …………………………….
3. Ntchito ya mankhwala amenewa ndi chiyani (14) for? (i) ……………………………… (ii) …………………………….…………
4. Pali mavuto aliwonse amene abwela mthupi mwanu chifukwa cha mankhwala a sugar?

Eya Ayi

1. Ngati eya, ndi mavuto ati amene abwela mthupi mwanu chifukwa cha mankhwala a sugar? (i) …………………………………….. (ii) ………..................................................

**Kagwiritsidwe ntchito kamakwala azisamba kumatenda a kuthamanga magazi?**

1. Munamwako makhwala a zitsamba kumatenda a kuthamanga magazi kapena mtima?

Eya Ayi

1. Ngati Eya, ndi mtengo wango wanji ndi mbali iti imene munagwiritsako ntchito?

(i) ………………… …..(ii) ……………………………. (iii) …………………………………

1. Mwagwiritsa ntchito mankhwala azisamba nthawi yayitali bwanji?

Panopa Osapitirira miyezi 6 Pakati pa myezi 6 ndi chaka

Kupitilira chaka

1. Amachokera kuti mankwala azisamba amene mumamwawo? Pharmacy

Kwasingánga Ma venda Anzanu Kumunda/Kutchile Kwina……………………………………….

1. Pali vuto lina lililonse limene linabwera mthupi lanu chifukwa chogwiritsa mankhwala a zitsamba? Eya Ayi
2. Ngati Eya, Ndimavuto anji amene anabwera mthupi lanu chifukwa chogwiritsa mankhwala a zitsamba? (i) ………………………………………. (ii) …………………………………
3. Munayamba mwamwera limodzi mankhwala a zitsamba ndi mankhwala a BP? Eya Ayi
4. Kuphatikiza mankhwala a BP ndi zitsamba (i) Zimathandiza (ii) Zizithandiza

(iii) Zimabweretsa mavuto (iv) Zindikudziwa (v) Zina ………………..

**Kagwiritsidwe ntchito kamankhwala azitsamba kumatenda a shuga**

1. Munamwako makhwala a zitsamba kumatenda a kuthamanga a sugar?

Eya Ayi

1. Ngati Eya, ndi mtengo wango wanji ndi mbali iti imene munagwiritsako ntchito?

(i) ………………… …..(ii) ……………………………. (iii) …………………………………

1. Mwagwiritsa ntchito mankhwala azisamba nthawi yayitali bwanji?

Panopa Osapitirira miyezi 6 Pakati pa myezi 6 ndi chaka

Kupitilira chaka

1. Amachokera kuti mankhwala azisamba amene mumamwawo? Pharmacy

Kwasingánga Ma venda Anzanu Kumunda/Kutchile Kwina……………………………………….

1. Pali vuto lina lililonse limene linabwera mthupi lanu chifukwa chogwiritsa mankhwala a zitsamba? Eya Ayi
2. Ngati Eya, Ndimavuto anji amene anabwera mthupi lanu chifukwa chogwiritsa mankhwala a zitsamba? (i) ………………………………………. (ii) …………………………………
3. Munayamba mwamwera pamodzi mankhwala a zitsamba ndi mankhwala a sugar? Eya Ayi
4. Kuphatikiza mankhwala a suagar ndi azitsamba (i) Zimathandiza (ii) Zizithandiza

(iii) Zimabweretsa mavuto (iv) Zindikudziwa (v) Zina ………………..

_________________________________________________________________

**END OF QUESTIONNAIRE**
